# Antibiotic susceptibility pattern in urinary isolates of gram negative bacilli with special reference to AmpC β-lactamase in a tertiary care hospital

**DOI:** 10.4103/0974-7796.62915

**Published:** 2010

**Authors:** Mitesh H. Patel, Grishma R. Trivedi, Sachin M. Patel, Mahendra M. Vegad

**Affiliations:** Department of Microbiology, B J Medical College, Ahmedabad-380 016, India

**Keywords:** AmpC β-lactamase, β-lactamase, Inhibitor based method

## Abstract

**Introduction:**

Resistance to higher antimicrobial agent is commonly seen in gram negative bacilli. This issue is a challenging problem to the medical practitioners in addition to it is financial impact on the health care system.

**Objectives:**

To document the prevalence of multi drug resistant gram negative bacilli isolated from urine of patients attending the Urology Department of Tertiary care Hospital of western India in year 2008.

**Results:**

Out of total 328 isolates, 118 (35.98%) *E.coli*, 72 (21.95 %) *Klebsiella*, 64 (19.51%) *Pseudomonas aeruginosa*, 30 (9.15%) *Acinetobacter*, 18 (5.49%) *Proteus vulgaris*, 18 (5.49%) *Proteus mirabilis*, 6 (1.83%) *Providencia rettgerii*, 2 (0.61%) *Citrobacter freundii*. Out of these isolates, 228 (69.51%) were β-lactamase positive, while 100 (30.51%) were β-lactamase negative. Out of 228 β-lactamase positive, 104 (45.61%) were AmpC β-lactamase positive.

**Conclusions:**

Stringent protocol such as Antibiotic policy and Hospital infection control program are mandatory to curb these microbes in a tertiary care hospital.

## INTRODUCTION

Bacteria cause infectious diseases and antimicrobial agents have been developed to combat the severity and spread of many of these diseases. In last decade bacteria emerged with new forms of virulence and new patterns of resistance to antimicrobial agents. The emergence of resistance to such drugs is a natural biological phenomenon.[1] The use of antibiotics for any infection causes a "selective pressure" on bacterial populations. Therefore, resistant mutants emerge under selection pressure and the mutants can flourish. Therefore, it becomes important to diagnose the resistance pattern of bugs.

### β-lactam antibiotic and β-lactamase enzymes

The failure of empirical therapy is a frequent and common problem in urinary tract infection. Therefore, we conducted a prospective clinical base study in 2008 to determine resistant profile of gram negative bacteria isolated from urine. β-lactam antimicrobial agents are one of the most common agents used to treat various infectious diseases and the most common agents to which gram negative bacilli have developed resistance.[2]

As gram negative bacilli are the most common isolates from the various clinical specimens and multidrug resistant gram negative bacilli are responsible for an increasing numbers of serious nosocomial and community-acquired infections. The mechanism of such resistance in bugs are diverse and the resistance pattern of β-lactam antibiotics is one of the most studied topics in the last few decades although it seems inadequate.

β-lactamases are the main cause of bacterial resistance to penicillin and cephalosporin. The important question here is which β-lactamase enzyme is produced by gram negative bacilli and its method of detection, which ultimately helps in deciding the line of antibiotic therapy.[3]

In many cases empirically prescribed antibiotics are going to fail to eliminate urinary pathogens. Among multidrug resistant gram negative bacteria, Extended spectrum β-lactamase (ESBL) producing *E.coli, Klebsiella* etc., Metallo-β-lactamases (MBLs) producing *Pseudomonas aeruginosa, Acinetobacter*, etc., Inducible β-lactamase (AmpC β-lactamase) in SPICE group (S – *Serattia*, P – *Pseudomonas aeruginosa*, I – Indole positive Proteus, C – *Cirtobacter*, E – *Enterobacter*), are prevalent as nosocomial pathogens. Infections by these drug resistant strains are associated with significant morbidity and mortality. Multi drug resistant organisms worsen the situation in this era of hospital acquired infections and thus limit therapeutic options, mainly with carbapenem and BL/BLI (β-lactam /β-lactamase inhibitor) group.

β-lactamase inhibitor resemble β-lactam molecules but they have very weak antibacterial action. They bind to the β-lactamases either reversibly or irreversibly and protect the β-lactam ring from being hydrolyzed. Commonly used β-lactamase inhibitors are Clavulanic acid, Sulbactam and Tazobactam. Clavulanic acid and Tazobactam are superior to Sulbactam. All three inhibitors are effective against Staphylococcal Penicillinase and have variable effectiveness against the β-lactamase enzymes of GNBs.

### Types and it nomenclature of AmpC β-lactamase:

1. Constitutive and inducible AmpC β-lactamase:
  - Constitutive – AmpC β-lactamases usually present at a low level in gram negative bacilli.
  - Inducible – Only in presence of inducer β-lactum drug, AmpC β-lactamase production takes place. Inducers for AmpC β-lactamase production - Cefoxitin (Strong inducer), Clavulanic acid, Imipenem
2. Chromosomal and Plasmid mediated AmpC β-lactamase:
  - Chromosomal – Because of mutation certain bacteria overproduce this enzyme, initially AmpC β- lactamases are presumed to be chromosomal.
  - Plasmid mediated - Subsequently, plasmid mediated AmpC- β-lactamases have been discovered.

In ESBL producing bacteria, all the member of penicillin, cephalosporin and monobactum (Aztreonam) are ineffective, irrespective of their *in vitro* results in culture and sensitivity report. Therefore, only BL/BLI combinations (e.g., Ampicillin + Sulbactum, Cefoperazone + Sulbactum, Piperacillin + Tazobactum, Ticarcillin + Clavulanic acid, etc.) and carbapenems are choice of drugs. While in Metallo-β-blactamase (MBL) producing bacteria, all β-lactam are resistant except Monobactum. In AmpC β-lactamase producing bacteria, if the combination drug shows resistance for a particular strain, then there is likely to be AmpC β-lactamase production. So along with penicillin, cephalosporin and Monobactum, BL/BLI combination drugs are also not effective. So choice remain only with carbapenem group.

## MATERIALS AND METHODS

The purpose of this study was to evaluate the antibiotic susceptibility pattern (antibiogram) of gram negative bacilli in various clinical specimens, to find out prevalence of β-lactamase enzyme production with special reference of AmpC β-lactamase in a tertiary care hospital. So as per the prevalence of various β-lactamase enzymes, urologists can prescribe most successful empirical antibiotic to eliminate urinary pathogens, before exact antibiogram is available for a particular isolates from a urine culture of patient. All patients had received antibiotic for various period ranging from seven days to thirty days, depending upon clinical response to antibiotics by various patients. Antibiotics were stopped after patients become asymptomatic. It was confirmed by absence of pus cells, RBCs in microscopic examination and absence of bacteria in gram stain of urine.

### Selection of patients

The present study comprising of 500 Patients having one or more than one urinary symptoms, like burning during micturition, fever, pyuria, frequency of urine, dysuria, hematuria, flank pain, suprapubic discomfort, etc., were selected. Patients have various precipitating factors like stricture, stone, diverticula, etc. Mid stream urine sample in early morning was collected in wide mouth sterile container. Female patient should be instructed to cleanse the area around the urethral opening with clean water, dry the area, and collect the urine with the labia held apart. In patient's having urinary catheter, urine was collected after clamping it for ten minutes. All urine samples were examined by routine microscopic examination by wet mount of urine sediment after centrifuging urine for ten minutes at 1000 rpm (revolution per minute). Presence of pus cells, RBCs, epithelial cells, casts and crystals are noted as supportive findings of urinary infection. Simultaneously all urine were cultured over routine culture media; Blood agar and Mac conkey agar, and incubated at 37 degree centigrade for two consecutive overnight for growth of any pyogenic bacteria. Out of these 500 samples, 328 samples show growth of gram negative bacilli, while remaining samples were either negative for bacterial growth or show growth other than gram negative bacilli. All 328 gram negative bacillary isolates were identified up to species level by standard biochemical reactions and antibiotic susceptibility was performed by Kirby Bauer method as per CLSI (Clinical and Laboratory Standard Institute, formerly NCCLS) guidelines.[4]

### Selection of antibiotic for testing susceptibility

The antibiotics tested for each gram negative isolates were Ampicillin-sulbactam (20 μg), Cefuroxime (30 μg), Ceftriaxone (30 μg), Cefoxitin (30 μg), Cefepime (30 μg), Levofloxacin (5 μg), Amikacin (30 μg), Cefoperazone-Sulbactam (105 μg) and Imipenem (10 μg). (Himedia Laboratory, Mumbai)

### Confirmation of the production of β-lactamase enzyme

The isolates were screened for β-lactamase enzyme production with Nitrocefin (chromogenic cephalosporin) test (Cefinase, B.D Microbiology Systems).[5‐7]

### Detection of AmpC β-lactamase

This was done by using Inhibitor based method. As cefoxitin is a potent inducer of AmpC β-lactamase, So when cefoxitin is tested against bacterial strain, bacteria start to produce AmpC β-lactamase. So cefoxitin show resistance against particular bacterial strain. Boronic acid have characteristic that it inactivate AmpC β-lactamase. So disk containing cefoxitin and boronic acid is tested against bacterial strain, boronic acid inactivate AmpC β-lactamase, so cefoxitin shows zone of inhibition against particular bacterial strain. So it indicates induction of AmpC β-lactamase in particular bacterial strain.[8,9]

### Preparation of cefoxitin-boronic acid disks

One hundred twenty milligram of phenylboronic acid (Sigma Aldrich) was dissolved in three ml of dimethyl sulfoxide. Three milliliter of sterile distilled water was added to this solution. Twenty micro liter (400 μg) of the stock solution was dispensed onto disks containing 30 μg of cefoxitin disks. Disks were allowed to dry for 30 min and then used for the test.

### Method of detection

Lawn culture of the isolate was done on Muller Hinton Agar. Disk containing 30 μg of cefoxitin and another disk containing 30 μg of cefoxitin with 400 μg of boronic acid were placed on the agar at a distance of 30 mm. Inoculated plates were incubated overnight at 37°C.

### Interpretation

An organism exhibiting a zone diameter around the disk containing cefoxitin -boronic acid five mm or greater than zone diameter around the disk containing cefoxitin alone was considered as an AmpC producer.[10,11]

## RESULTS

Following bacteria were confirmed up to species level. Out of 328 gram negative bacilli, *E. coli* was most common isolates. (n = 118) [Table 1]

**Table 1:** Gram negative bacilli isolated during the study

*Gram Negative Bacilli*	Frequency	
	Number(328)	Percentage
*E. coli*	118	35.98
*Klebsiella pneumonia*	72	21.95
*Pseudomonas aeruginosa*	64	19.51
*Acinetobacter baumanii*	30	9.15
*Proteus mirabilis*	18	5.49
*Proteus vulgaris*	18	5.49
*Providencia rettgerii*	6	1.83
*Citrobacter freundii*	2	0.61

### Antibiogram

Out of 328, only ten isolates were sensitive to the panel of antibiotics tested. Rest 318 isolates showed resistance to at least one antibiotic. All isolates were 100% sensitive to Imipenem [Tables 2 and 3].

So from selected antibiotics tested, most effective antibiotic is imipenem and least one is cefuroxime and cefoxitin (2nd generation cephalosporin). Levofloxacin and amikacin are showing less susceptibility as compared to BL/BLI group.

**Table 2:** Antibiotic resistance pattern of gram negative bacilli

Antibiotics →	Ampicillin + Sulbactum	Cefuroxime	Cefoxitin	Ceftriaxone	Cefepime	Cefoperozone + Sulbactum	Imipenem	Amikacin	Levofloxacin
Isolates ↓									
*E.coli*	22	98	80	80	22	12	0	32	22
*(n =118)*	18.64	83.05	67.79	67.79	18.64	10.17	0	27.12	18.64
*(%)*									
*Klebsiella*	26	66	58	58	16	4	0	36	14
*(n =72)*	36.11	91.67	80.55	80.55	22.22	5.55	0	50	19.44
*(%)*									
*Pseudomonas*	32	34	32	32	10	0	0	30	26
*(n =64)*	50	53.12	50	50	15.63	0	0	46.88	40.63
*(%)*									
*Acinetobacter*	10	22	18	18	4	2	0	16	6
*(n =30)*	33.33	73.33	60	60	13.33	6.67	0	53.33	20
*(%)*									
Proteus	8	18	16	18	8	0	0	6	0
*mirabilis(n = 18)*	44.44	100	88.88	100	44.44	0	0	33.33	0
*(%)*									
*Proteus vulgaris*	6	16	10	14	4	0	0	6	0
*(n =18)*	33.33	88.88	55.55	77.77	22.22	0	0	33.33	0
*(%)*									
*Providencia*	2	4	4	4	2	0	0	4	0
*Rettgerii(n = 6)*	33.33	66.67	66.67	66.67	33.33	0	0	66.67	0
*(%)*									
*Citrobacter*	2	2	2	2	0	0	0	2	2
*freundii(n = 2)*	100	100	100	100	0	0	0	100	100
*(%)*									

**Table 3:** Overall resistance pattern

Antibiotics tested	Isolates showing resistance	Percentage of resistance
Ampicillin-sulbactam	108	32.96
Cefuroxime	268	81.70
Cefoxitin	220	67.07
Ceftriaxone	226	68.90
Cefepime	60	18.29
Cefoperazone-sulbactam	18	5.48
Imipenem	0	0
Amikacin	132	40.24
Levofloxacin	70	21.34

Out of total 328 isolates, 228 (69.51%) were positive in Nitrocefin test – a test to detect the β-lactamase enzyme production, while 100 (30.49%) isolates were negative for the same.

Out of total 228 β-lactamase enzyme producing isolates 104 (45.61%) were positive for AmpC β-lactamase enzyme production, while 124 (54.39%) isolates were negative for the same. Out of 104 AmpC positive gram negative bacteria, 38 were *E. coli*, 26 were *Klebsiella pneumonia*, 18 were *Pseudomonas aeruginosa*, 12 were *Acinetobacter baumanii*, Two were *Proteus mirabilis*, Six were *Proteus vulgaris* and Two were *Providencia rettgerii*.

## DISCUSSION

Related to many other studies, here much higher resistance pattern was observed.[12‐14] The above difference may be due to the geographic variations that were observed in the different strains of gram negative bacilli. In present study, 69.51 % of gram negative isolate producing β-lactamase enzymes, so they are resistant to β-lactam group, but here simultaneously AmpC β-lactamase enzyme is also detected.[15,16]

As compared to 45.61% AmpC β-lactamase prevalence in present study, Three other studies were reported 8, 43 and 47 percent prevalence of AmpC β-lactamase.[8,17,18] It means that only carbapenem is remain choice in such types of resistant bugs.

Also, in this study, multidrug resistance was observed. The possible mechanism of resistance may be: 1) Intracellular degradation of antibiotic 2) Hyper production of chromosomal class C enzymes, 3) Presence of multidrug efflux system, 4) Low outer membrane permeability, 5) Resistance factor with Resistance transfer factor in plasmid. 6) Poor binding with cell surface receptor

This study helps in making decision regarding empirical prescription of antibiotic in urinary tract infection until culture report is available. So BL/BLI or carbapenems are the choice of drugs for empirical treatment of urinary tract infection by gram negative bacteria, until presence of β-lactamase is ruled out and exact sensitivity report is available for a particular patient. Therefore, it suggests the valuable therapeutic can be obtained after performing the gram stain.

## CONCLUSION

In the era where a resistant gram negative bacilli strain emerges, there is not necessarily a new "wonder drug" against it is ready on the shelf in pharmacy. Most threatening of all gram negative bacteria is that, they have "accumulated" resistance genes to virtually all currently available drugs and have the potential to cause untreatable infections, thus raising the spectrum of a post antibiotic era. Even if the pharmaceutical industry were to step up efforts to develop new drugs immediately, current trends suggest that some diseases will have no effective therapies.

The result of present study shows higher rate of resistance in a tertiary care hospital, which is the result of the irrational use of antibiotics. Irrational use of antibiotics bring us at a point, as frightening as the pre antibiotic era for patients infected with multidrug-resistant gram negative bacteria, where there is no magic bullet available.

The diagnosis of the infecting organism up to species level along with the mechanism of resistance helps in the judicious use of chemotherapeutic agents effective against them, which will withdraw the selection pressure and resistant bacteria will no longer have survival advantage in such settings.

The inhibitor based confirmatory method, used in this study, proved helpful to detect AmpC β-lactamase enzymes, the most common mechanism of resistance in gram negative bacilli after the ESBL (Extended spectrum β-lactamase) production. When both enzymes are present together can mask the phenotype of each other.[19] So the detection becomes very difficult. The inhibitor based method detects when both enzymes are present simultaneously and it is easy to perform. The results are helpful to formulate an empirical therapy in different clinical situations.

A concerted effort on the part of academic researchers and their institutions, industry, and government is crucial if humans are to maintain the upper hand in this battle against bacteria - a fight with global consequences. Otherwise, we may reach to era, where "Bad bugs, there is no drugs".
